# First population norms for the EQ-5D-3L in the Russian Federation

**DOI:** 10.1371/journal.pone.0263816

**Published:** 2022-03-29

**Authors:** Alina Khabibullina, Ekaterina Aleksandrova, Christopher J. Gerry, Vasily Vlassov

**Affiliations:** 1 International Centre for Health Economics, Management and Policy, National Research University Higher School of Economics, St. Petersburg, Russian Federation; 2 Oxford School of Global and Area Studies, University of Oxford, Oxford, England; 3 Department of Health Care Administration and Economics, National Research University Higher School of Economics, Moscow, Russian Federation; PLOS: Public Library of Science, UNITED KINGDOM

## Abstract

**Purpose:**

The EQ–5D survey instrument is routinely applied to general and patient specific populations in many countries, as a means of measuring Health Related Quality of Life (HRQOL) and/or informing Health Technology Assessment. The instrument is the subject of growing interest in the Russian Federation, as too is Health Technology Assessment. This research is the first to systematically present the EQ–5D–3L nationally representative population norms and to examine the socioeconomic and socio-demographic characteristics of the instrument among a representative sample of the Russian population.

**Methods:**

Based on a nationally representative health and well-being survey of the Russian population, conducted in November 2017, we establish the descriptive results, including the EQ-VAS and the EQ-5D Index, by age and gender, examine the correspondence between the EQ–5D health classifications and the separate EQ-VAS scores, and draw on a set of augmented logistic regressions to evaluate the association between the presence of problems in each dimension and various socio-economic and health-related characteristics.

**Results:**

We find strong evidence that the EQ-5D instrument is sensitive to underlying observed and latent health experiences, that it mirrors many of the characteristics familiar from other settings but that there are Russian specificities which merit further research, particularly with respect to the anxiety/depression dimension of the instrument.

**Conclusion:**

This research represents an important landmark for HRQOL studies in Russia as well as for the prospects of continuing to develop the scholarly and practical infrastructure necessary for Russian Health Technology Assessment to advance.

## Introduction

In recent decades health-related quality of life (HRQOL) has become an increasingly important additional outcome of clinical trials [1,2], public health interventions [3,4] and health care more broadly [5]. Individual HRQOL is closely connected to social, economic and personal circumstances and its measurement is therefore complex [6,7]. Diverse instruments have evolved in response. The EQ-5D instrument, developed by the EuroQol Group in the late 1980s, is a standardized HRQOL questionnaire intended to provide a simple, generic measure of health for clinical and social appraisal [8,9]. The EQ-5D questionnaire is now available in more than 170 translated versions; is used in clinical trials, observational studies and population health studies; and across academia, government, the pharmaceutical industry, as well as by hospitals and clinics [10]. It is the most frequently utilised tool for evaluating the efficacy of (potential) health care interventions in cost-utility analyses.

The EQ-5D instrument comprises a descriptive system that measures current HRQOL across five dimensions (mobility, self-care, usual activities, pain/discomfort, and anxiety/depression). In each dimension a self-reported level of HRQOL is chosen from among either three levels (3L) or five levels (5L) indicating the severity of health problems in that dimension. In addition, respondents are asked to assess their own “health state today” by positioning themselves on a visual analogue scale (EQ-VAS) on which 0 represents the worst imaginable state and 100 the best imaginable state. Finally, the responses to the descriptive system provide a set of scores which can (optionally) be aggregated to a single index value (EQ-5D Index), using utility tariffs believed to reflect the general population preferences between different HRQOL outcomes. A number of time/country specific tariffs exist, which can provide distinct EQ-5D indices to be used for estimating the Quality-Adjusted Life Years (QALYs) used in some Health Technology Assessment [11].

The EQ–5D instrument has proven to be reliable and is now routinely applied to general and patient specific populations, in many countries [12,13], with Russia no exception [14–17]. These EQ–5D surveys are informative in providing new data on HRQOL within general and patient specific populations. For Russia, this provides the potential for scientists to identify the true burden of disease in particular patient populations or to inform clinical decision making more directly through scoring the descriptive results with utility tariffs and producing QALYs. In the latter case, the scientific and practical foundations for gathering population preferences are in their infancy [18,19] but, very promisingly, systematic efforts to establish an EQ-5D-3L value set for Russia have recently begun [20]. In the former case, despite a recent presentation of Moscow norms for the EQ-5D-5L [21], Russia lacks the requisite nationally representative frame of reference or *population norms* necessary to interpret an individual’s descriptive scores relative to the scores of others. While the equivalent population norms for the EQ-5D, accounting for socio-demographic characteristics, are available for many countries [22], until now they do not exist for Russia. The primary contribution of this paper is therefore to directly describe health-related quality of life in Russia, across a representative population, using the EQ-5D-3L instrument. In doing so, we provide the first aggregated insights into the socioeconomic and socio-demographic characteristics of this HRQOL measure among a survey representative of the Russian population. In combination with the ongoing work of a Moscow-based team of researchers [20] this will help consolidate progress in Russia towards consistent and comparative measurement of HRQOL, more widespread adoption of cost-utility analysis and the promotion of Health Technology Assessment.

## Methods

### Data

The population norms presented in this paper are based on a November 2017 population survey of self-reported HRQOL outcomes and behaviours conducted by the Levada Center–an independent Russian sociological polling organisation established in 2003. The survey was conducted as part of a frequent ‘omnibus’ series of Levada surveys funded by the National Research University Higher School of Economics (HSE) to obtain regular population data on socioeconomic and demographic characteristics. The survey is conducted through voluntary face-to-face interviews undertaken by professional interviewers, following best international practice in ethical protocol. The data are drawn from a nationwide, multistage probability sample of 137 settlement units, comprising of 97 urban and 40 rural areas. The sample covers all Federal Russian districts and five settlement types in each macro-region in proportion to the size of the local population aged 18 and over. The secondary sampling units are chosen from the lists of polling stations in urban settlements and villages in rural districts. Households are selected through the random route method and individual respondents are identified according to survey quotas that control for gender, age, and education level with respect to the 2010 Russian Population Census and current Russian federal state statistics data. All Levada surveys are further subject to additional multi-level quality controls. These include cross-checking route lists, interviewer reliability and quality and data coding/input protocol. Full details of the study design are available from the Levada Center [23]. The original, raw data with identifiers is accessed only by the Levada Center according to their approved data handling and management practices. The cleaned data, released to the researchers, is fully anonymised and re-coded so as to contain no identifying information and so that full anonymity is guaranteed. The full sample of 1,565 adults aged 18 years and above, is representative at both the national level and at the Federal District level (excluding the North Caucasus district, which is not surveyed) adjusting for the size of settlement, the residence type (urban/rural), age and gender.

The survey itself includes a standard set of omnibus questions covering gender, age, marital status, number of children, household size, education, occupation, income, region and size of town. In addition to these core questions, we incorporated a set of questions (S1 and S2 Files) specifically relating to the respondents HRQOL, including asking them: (a) to evaluate their health on a 5-category scale of self-assessed health (SAH); (b) whether they have particular chronic diseases; (c) whether they suffer from depression and anxiety; (d) to assess their health relative to others of the same age and gender; and (e) to report their official disability status.

### HRQOL instrument

In addition, we included the EQ-5D instrument. Respondents were asked to evaluate their current health status using the Russian version of the EQ–5D–3L questionnaire to which we made several important linguistic corrections, in line with earlier literature and approved by the EuroQoL group [19]. The three level (no problem, some problems, severe problems) instrument, in combination with the five dimensions gives rise to 243 possible unique health states. In the absence of a set of Russian health state tariffs (preference weights), we use the United Kingdom (UK) value set [24] for generating the EQ-5D Index for Russia. We augmented the EQ-5D instrument with the addition of a follow up question requiring respondents to consider the extent to which they had taken into account their mental health when providing their EQ–VAS rating.

### Analysis

Data analysis was carried out using the Stata software, version 14.0 (StataCorp. 2015. Stata Statistical Software: Release 14. College Station, TX: StataCorp LP). We first describe the self-reported EQ–5D–3L profiles and mean values for the EQ–VAS and the EQ-5D index, by age and gender sub-groups. As is usual in EQ-5D studies, we examine the correspondence between the EQ–5D health classifications and the separate EQ-VAS scores to seek to understand the contribution of each dimension to overall HRQOL [25]. Observed cross-group differences in mean EQ-VAS and EQ-5D index values are tested for the overall sample with the non-parametric Kruskal-Wallis (two groups) and Mann-Whitney (multiple groups) tests and logistic regression is then used to evaluate the association (odds ratios) between the presence of problems in each dimension and various socio-economic and health-related characteristics detailed above and summarised in Table 1. The dependent variables in the logistic regressions are the EQ–5D dimensions dichotomized according to the presence of reported problems, or not. We estimate a baseline set of regressions, in which the independent variables comprise of age, gender, education group, marital status, area of residence and an indicator of deprivation [26–28]. We then draw on the supplementary health-related data concerning chronic diseases, disability status, mental health and general self-assessed health for a second–augmented–set of regressions, which are presented in Table 6.

**Table 1:** Participants’ characteristics.

Sample	Total	Males	Females
	n = 1,565	n = 712	n = 853
Age in years, mean (SD)	46.1 (16.8)	44.0 (16.2)	47.8 (17.1)
**Age category, %**			
18–24	9.8	11.0	8.8
–34	21.2	23.9	19.0
35–44	18.9	20.4	17.6
45–54	15.8	15.7	15.8
55–64	18.1	15.9	19.9
65–74	11.0	9.8	12.0
>75	5.3	3.4	6.9
**Highest education level, %**			
Primary	24.4	25.8	23.2
Secondary	45.2	45.7	44.9
Higher	30.4	28.5	31.9
**Marital status, %**			
Single	13.7	18.0	10.2
Married	66.9	72.2	62.5
Divorced	8.0	5.9	9.7
Widowed	11.4	3.9	17.6
**Not able to afford food and clothes, %**			
No	79.3	82.7	76.4
Yes	20.7	17.3	23.6
**Residential area, %**			
Rural	24.8	25.8	23.9
Urban	75.2	74.2	76.1
**Whether has disability, %**			
No	90.4	91.4	89.6
Yes	9.6	8.6	10.4
**Negative feelings (blue mood), %**			
No	71.3	77.1	66.5
Yes	28.7	22.9	33.5
**Has chronic disease, %**			
None	54.8	63.6	47.4
Heart disease	13.8	10.3	16.8
Lung disease	2.8	2.7	2.9
Liver disease	3.2	2.5	3.7
Kidney disease	3.9	3.1	4.6
Gastroenteritis	8.6	6.0	10.8
Allergy disease	3.7	1.1	5.7
Ear, nose or throat disease	2.5	1.7	3.2
Cancer	0.5		0.8
Spinal disease	7.0	5.5	8.2
Other chronic diseases	16.2	13.5	18.4
**Number of chronic conditions, %**			
None	54.8	63.6	47.4
One	33.8	29.6	37.3
Two	7.4	4.6	9.7
Three or more	4.0	2.1	5.6
**Self-assessed health, %**			
Bad	11.4	9.2	13.2
Average (neither bad nor good)	47.7	42.6	52.0
Good	40.9	48.2	34.8
**Health relative to similar age/gender, %**			
Worse	10.8	8.4	12.9
Same	62.0	59.6	64.0
Better	27.2	32.0	23.1
**Fail to account for mental health in reporting VAS**			
Yes	32.7	34.3	31.3
No	67.3	65.7	68.7

## Results

The socio-demographic characteristics of the respondents (see Table 1) are consistent with those of the general population of Russia [29] and with those reported in the most respected national population survey–the Russia Longitudinal Monitoring Survey Higher School of Economics (RLMS-HSE) [30]. As is often the case with Russian household surveys, there is a slight over-sampling of females (55%) which gives rise to a corresponding oversampling of the elderly (who are disproportionately female in some regions due to the high mortality rates of Russian males). The mean age of women is 47.8 (SD:17.1), vs 44 (SD:16.1) for males. The majority of the sample are married (72.2% males; 62.5% females), but more males are single (18% vs 10.2%) and more females are widowed (17.6% vs 3.9%) and divorced (9.7% vs 5.9%). The educational attainments reflect that the sample is mostly well-educated, but only slightly more so than the general population, with approximately two-thirds having achieved either college or university education.

Before turning to the EQ-5D instrument, some brief comments on the supplementary health questions. We find that 22.9% of males and 33.5% of females report suffering from ‘blue mood’ or anxiety, while, interestingly, almost one-third of all respondents claim not to take into consideration their mental well-being when reporting their EQ–VAS score. This merits further attention in future research. Thirty per cent of males and just under a quarter of females feel that their relative health compares favourably with others of their age and gender. Around one-third of males and approximately one-half of women report having at least one chronic disease, the most common of which is heart disease (10.3% males, 16.8% females). These data are consistent with those reported in studies based on the RLMS-HSE data, which also discuss the apparent paradox of males reporting superior health to that of females [31].

The mean EQ-VAS scores, by socio-demographic and health-related characteristics, are summarised in Table 2. The mean EQ-VAS score is 70.5 (SD: 21,1) with a statistically higher score for males (73.9) compared to females (67.6) and lower scores among higher age groups (p < 0.0001). Divorced or widowed participants have significantly lower scores compared to other marital categories (p < 0.0001) while those who are poor also report significantly lower EQ-VAS scores on average (59.3 vs 73.4). There are also notable differences in EQ-VAS scores in terms of education attainment, place of residence, and SAH. Similarly, people suffering from chronic diseases, having a disability, or experiencing feelings of blue mood report significantly lower EQ-VAS scores than their counterparts (p < 0.0001). The EQ-VAS scores for those reporting no chronic illnesses are approximately 150% those of respondents reporting two or more chronic conditions. Indeed, the number of reported chronic illnesses is a very good predictor of HRQOL. These findings are consistent with earlier literature based on the RLMS-HSE [32].

**Table 2:** Mean EQ-VAS scores by different characteristics.

Sample	Total			Males			Females		
	Freq.	Mean	CI 95%	Freq.	Mean	CI 95%	Freq.	Mean	CI 95%
	100	70.5	69.5; 71.6	45.5	73.9	72.4; 75.5	54.5	67.6	66.2; 69.1
z = -6.282. p = 0.0000									
**Age category, % (***χ*^2^ = 562.241. p = 0.0001^a^)									
18–24	9.8	86.1	83.3; 88.9	11	87	82.8; 91.2	8.8	85.2	81.4; 89
25–34	21.2	82.3	80.6; 84.1	23.9	84	81.7; 86.4	19	80.5	77.9; 83.2
35–44	18.9	77.2	75.1; 79.3	20.4	79.3	76.1; 82.5	17.6	75.1	72.3; 77.9
45–54	15.8	67.9	65.5; 70.3	15.7	69.5	65.8; 73.3	15.8	66.5	63.3; 69.7
55–64	18.1	59.8	57.7; 61.8	15.9	62.6	59.3; 65.9	19.9	57.9	55.3; 60.5
65–74	11	53.6	51.2; 56	9.8	55.2	51.8; 58.7	12	52.5	49.2; 55.9
>75	5.3	50.2	46.3; 54	3.4	55.5	47.6; 63.4	6.9	48	43.6; 52.4
**Education level, %** (*χ*^2^ = 35.892. p = 0.0001)									
Primary	24.4	66.7	64.4; 68.9	25.8	69.8	66.6; 73	23.2	63.8	60.6; 67
Secondary	45.2	69.7	68.2; 71.2	45.7	73.6	71.3; 75.9	44.9	66.3	64.3; 68.3
Higher	30.4	74.8	73; 76.6	28.5	78.2	75.5; 80.8	31.9	72.3	69.9; 74.8
**Marital status, %** (*χ*^2^ = 214.201. p = 0.0001)									
Divorced	8	69.7	66.2; 73.2	5.9	76.1	70.4; 81.7	9.7	66.5	62.2; 70.7
Married	66.9	71.3	70; 72.5	72.2	72	70.2; 73.8	62.5	70.5	68.8; 72.2
Single	13.7	81.9	79.2; 84.6	18	85	81.8; 88.2	10.2	77.3	72.8; 81.9
Widowed	11.4	52.9	50.3; 55.5	3.9	55.4	46.9; 63.9	17.6	52.4	49.7; 55.2
**Not enough money for food and clothes, %** (z = 10.87. p = 0.0000)									
No	79.3	73.4	72.3; 74.6	82.7	75.8	74.1; 77.4	76.4	71.3	69.8; 72.9
Yes	20.7	59.3	56.9; 61.6	17.3	65.1	61.2; 69	23.6	55.7	52.9; 58.4
**Residential area, %** (z = 8.3. p = 0.0004))									
Rural	24.8	68.1	66; 70.2	25.8	72.3	69.3; 75.3	23.9	64.3	61.5; 67.1
Urban	75.2	71.3	70.1; 72.5	74.2	74.5	72.7; 76.3	76.1	68.7	67.1; 70.3
**Disability, %** (z = 12.222. p = 0.0000)									
None	90.4	72.8	71.7; 73.8	91.4	75.8	74.2; 77.3	89.6	70.1	68.8; 71.6
Disability	9.6	49.1	45.9; 52.4	8.6	54	48.6; 59.5	10.4	45.8	41.9; 49.7
**Negative feelings (blue mood), %** (z = 14.1 p = 0.0000)									
No	71.3	75.2	74.1; 76.3	77.1	77.2	75.6; 78.9	66.5	73.3	71.7; 74.8
Yes	28.7	58.8	56.9; 60.8	22.9	62.9	59.7; 66.1	33.5	56.6	54.1; 59
**Self-assessed health, %** (*χ*^2^ = 575.5. p = 0.0001)									
Bad	11.4	47.9	45.3; 50.5	9.2	66.8	45.9; 54.8	13.2	63	43.3; 49.7
Average	47.7	64.6	63.3; 65.9	42.6	50.4	64.9; 68.8	52	46.5	61.3; 64.7
Good	40.9	83.7	82.4; 84.9	48.2	84.7	82.8; 86.5	34.8	82.6	80.9; 84.2
**Health relative to similar age/gender, %** (*χ*^2^ = 198. p = 0.0001)									
Worse	10.8	50.7	47.5; 53.9	8.4	56.6	50.9; 62.3	12.9	47.4	43.7; 51.2
Same	62	70.7	69.4; 72	59.6	72.8	70.9; 74.7	64	69.1	67.4; 70.8
Better	27.2	78.2	76.3; 80.2	32	81.2	78.4; 83.9	23.1	74.8	72.1; 77.5
**Number of chronic conditions, %** (*χ*^2^ = 456.212. p = 0.0001)									
None	54.8	77.2	76.1; 78.3	63.6	78.9	77.3; 80.6	47.4	75.4	73.8; 76.9
One	33.8	59.9	57.9; 62	29.6	60.2	57; 63.4	37.3	59.8	57.1; 62.5
Two	7.4	53.7	50.5; 57	4.6	58.4	54; 62.9	9.7	51.9	47.7; 56
Three or more	4	47	42.6; 51.4	2.1	48.3	37.3; 59.3	5.6	46.6	41.6; 51.5

Note

^a^p-values report differences in mean EQ-VAS values according to different characteristics of respondents for the overall sample.

Table 3 summarises the EQ–5D Index scores according to the different characteristics of respondents. The gender pattern observed for EQ-VAS is replicated over all five EQ–5D dimensions, though it is noteworthy that the ranking order of the difficulties experienced in each dimension is the same for men and women: that is, self-care, mobility, usual activities, pain and anxiety; from least to most problematic. The mean EQ-5D index is 0.84 (SD = 0.23) with males (0.87) reporting significantly higher scores than females (0.81). The means differed significantly (p < 0.0001) across all socio-demographic and health-related variables. Lower index scores were observed in higher age groups, lower education groups, and among the poor. Participants who were divorced or (particularly) widowed had the lowest index score compared to other marital categories. Similarly, people suffering from chronic diseases, feeling blue mood/anxiety or with any other health issues had significantly lower utility scores compared with their counterparts (p < 0.0001). These results are all in line with those for the EQ-VAS.

**Table 3:** Mean EQ–5D index by different characteristics.

Sample	Total			Males			Females		
	Freq.	Mean	CI 95%	Freq.	Mean	CI 95%	Freq.	Mean	CI 95%
	100	0.84	0.82; 0.85	45.5	0.87	0.85; 0.88	54.5	0.81	0.79; 0.82
z = -5.742. p = 0.0000									
**Age category, %** (*χ*^2^ = 502.4. p = 0.0001 ^a^)									
18–24	9.8	0.96	0.94;0.98	11	0.97	0.95;0.99	8.8	0.95	0.91;0.98
25–34	21.2	0.95	0.94;0.96	23.9	0.95	0.93;0.97	19	0.95	0.94;0.97
35–44	18.9	0.92	0.90;0.93	20.4	0.94	0.92;0.96	17.6	0.89	0.86;0.92
45–54	15.8	0.84	0.81;0.86	15.7	0.85	0.81;0.88	15.8	0.83	0.79;0.86
55–64	18.1	0.74	0.72;0.77	15.9	0.77	0.73;0.82	19.9	0.72	0.69;0.76
65–74	11	0.65	0.62;0.69	9.8	0.68	0.62;0.75	12	0.64	0.60;0.68
>75	5.3	0.56	0.49;0.62	3.4	0.66	0.54;0.77	6.9	0.52	0.44;0.60
**Education level, %** (*χ*^2^ = 29.039. p = 0.0001)									
Primary	24.4	0.80	0.77;0.82	25.8	0.86	0.79;0.86	23.2	0.77	0.73;0.81
Secondary	45.2	0.83	0.81;0.84	45.7	0.87	0.84;0.89	44.9	0.79	0.77;0.82
Higher	30.4	0.88	0.86;0.90	28.5	0.91	0.89;0.93	31.9	0.86	0.84;0.88
**Marital status, %** (*χ*^2^ = 201.043. p = 0.0001)									
Divorced	8	0.79	0.75;0.84	5.9	0.81	0.72;0.89	9.7	0.79	0.73;0.84
Married	66.9	0.86	0.85;0.87	72.2	0.87	0.86;0.89	62.5	0.85	0.83;0.87
Single	13.7	0.92	0.90;0.94	18	0.93	0.91;0.96	10.2	0.90	0.85;0.94
Widowed	11.4	0.62	0.58;0.67	3.9	0.60	0.47;0.74	17.6	0.63	0.58;0.67
**Not enough money for food and clothes, %** (z = 11.840. p = 0.0000)									
No	79.3	0.87	0.86;0.88	82.7	0.90	0.88;0.91	76.4	0.85	0.83;0.86
Yes	20.7	0.70	0.67;0.73	17.3	0.74	0.69;0.79	23.6	0.68	0.64;0.72
**Residential area, %** (z = -2.623. p = 0.0087)									
Rural	24.8	0.81	0.79;0.84	25.8	0.84	0.81;0.87	23.9	0.79	0.75;0.82
Urban	75.2	0.84	0.83;0.86	74.2	0.88	0.86;0.90	76.1	0.82	0.80;0.83
**Disability, %** (z = 15.163. p = 0.0000)									
None	90.4	0.87	0.86;0.88	91.4	0.90	0.88;0.91	89.6	0.84	0.83;0.86
Disability	9.6	0.54	0.49;0.58	8.6	0.57	0.49;0.64	10.4	0.51	0.45;0.58
**Negative feelings (blue mood), %** (z = 16.347 p = 0.0000)									
No	71.3	0.89	0.88;0.90	77.1	0.91	0.89;0.92	66.5	0.88	0.86;0.89
Yes	28.7	0.70	0.67;0.72	22.9	0.74	0.70;0.78	33.5	0.67	0.64;0.70
**Self-assessed health, %** (*χ*^2^ = 470.882. p = 0.0001)									
Bad	11.4	0.52	0.48;0.57	9.2	0.53	0.45;0.61	13.2	0.52	0.47;0.57
Average	47.7	0.82	0.81;0.83	42.6	0.85	0.83;0.87	52	0.8	0.78;0.82
Good	40.9	0.94	0.93;0.95	48.2	0.95	0.94;0.96	34.8	0.927	0.91;0.95
**Health relative to similar age/gender,** (*χ*^2^ = 241.491. p = 0.0001)									
Worse	10.8	0.57	0.52; 0.61	8.4	0.58	0.49; 0.67	12.9	0.57	0.52; 0.62
Same	62	0.86	0.84; 0.87	59.6	0.88	0.85; 0.89	64	0.84	0.82; 0.86
Better	27.2	0.90	0.88; 0.92	32	0.93	0.91; 0.94	23.1	0.87	0.83; 0.90
**Number of chronic conditions. %** (*χ*^2^ = 384.551. p = 0.0001)									
None	54.8	0.91	0.90;0.92	63.6	0.92	0.91;0.94	47.4	0.89	0.87;0.90
One	33.8	0.74	0.71;0.76	29.6	0.73	0.69;0.78	37.3	0.74	0.70;0.77
Two	7.4	0.65	0.59;0.70	4.6	0.65	0.54;0.75	9.7	0.64	0.58;0.70
Three or more	4	0.57	0.52;0.62	2.1	0.61	0.55;0.67	5.6	0.56	0.49;0.62

Note

^a^p-values report differences in mean EQ-VAS values according to different characteristics of respondents for the overall sample.

Turning to the EQ–5D descriptive profiles, 47.3% of respondents report moderate or severe problems (level 2 or 3) in at least one EQ–5D dimension, with the largest proportion of respondents (37.4%) reporting problems in the anxiety/depression component (31.04% male; 42.79% female). For comparison, 32.2% of respondents report pain/discomfort, 25.9% report mobility problems, 22.6% report restrictions on daily activities, while only 14% of respondents have difficulties taking care of themselves (S1 Table). We find a moderate relationship between the presence of negative feelings (blue mood) and the frequency of the moderate and severe problems in the “anxiety or depression” component and note that 69% of respondents who reported negative feelings (quite often, very often and always) also reported some problems in the anxiety/depression dimension of the EQ-5D instrument. Table 4 depicts each of the five EQ–5D domains according to the frequencies of each item response by gender, demonstrating again that women report significantly more problems in all five dimensions of the EQ–5D.

**Table 4:** Profiles of EQ–5D by gender, number of respondents.

	Dimension	Male			Female		
		1^a^	2	3	1	2	3
**D1**	**Mobility**	574 *80*.*62*^*b*^	130 *18*.*26*	8 *1*.*12*	585 *68*.*58*	263 *30*.*83*	5 *0*.*59*
**D2**	**Self-care**	641 *90*.*03*	68 *9*.*55*	3 *0*.*42*	705 *82*.*65*	141 *16*.*53*	7 *0*.*82*
**D3**	**Usual activity**	592 *83*.*15*	118 *16*.*57*	2 *0*.*28*	619 *72*.*57*	222 *26*.*03*	12 *1*.*41*
**D4**	**Pain/Discomfort**	527 *74*.*02*	177 *24*.*86*	8 *1*.*12*	534 *62*.*60*	301 *35*.*29*	18 *2*.*11*
**D5**	**Anxiety/Depression**	491 *68*.*96*	215 *30*.*20*	6 *0*.*84*	488 *57*.*21*	347 *40*.*68*	18 *2*.*11*

Note.

^a^1 –no problems; 2 –moderate problems; 3 –severe problems

^b^ the percentage of respondents is in italic.

**Fig 1:**
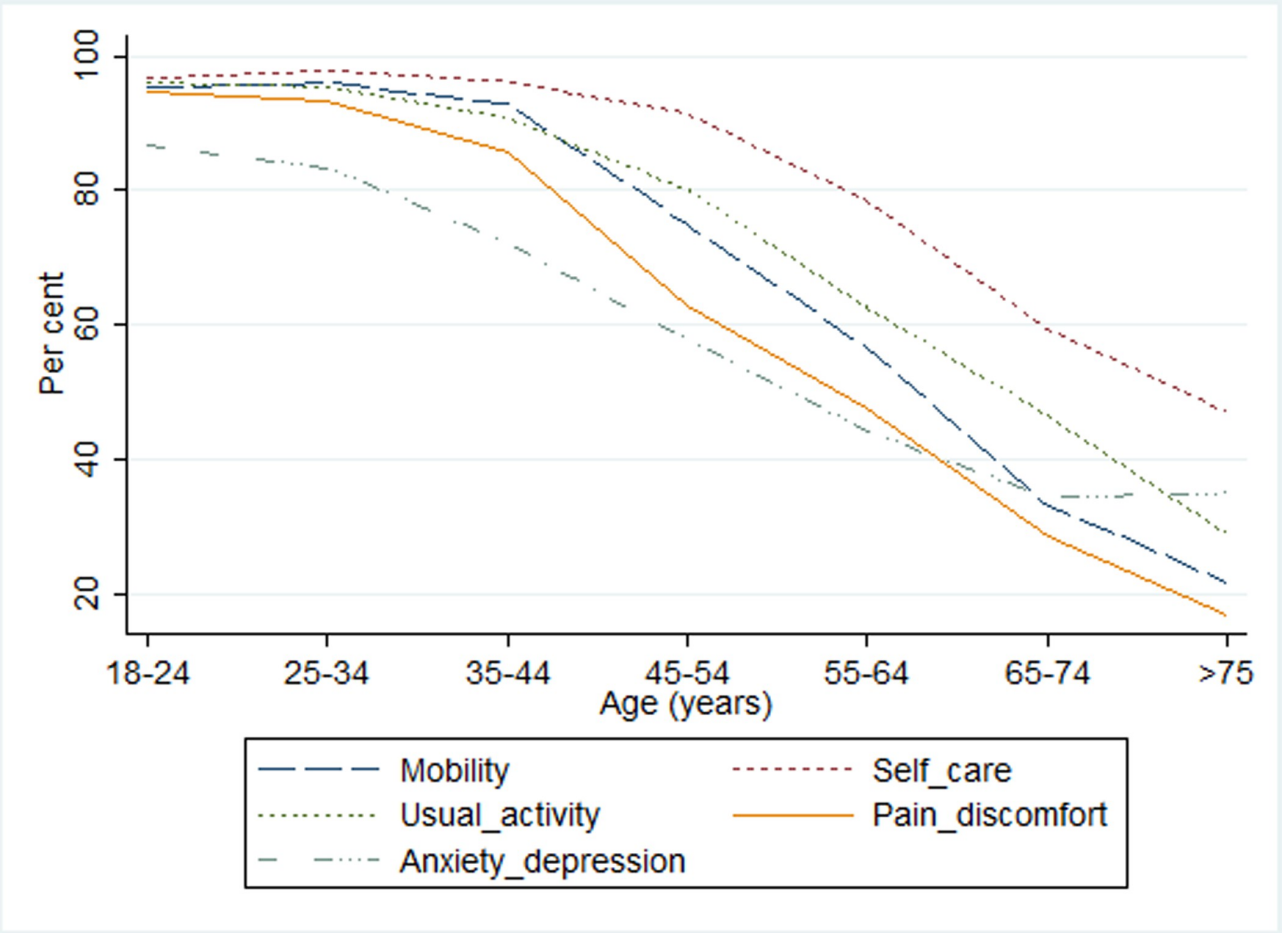
Per cent of respondents reporting level 1 (no problems) in each dimension.

The different rating scores for the age groups are presented in Fig 1 and S2 Table. A much higher proportion of elderly respondents, aged 75 and over (78.3%), report having mobility problems compared with those aged 18–24 (4.5%). The percentage of respondents reporting problems with self-care is generally low across all groups; however, almost half of elderly respondents (53%) report this problem compared with the youngest group (3.2%). The proportion of respondents having problems performing their usual activities is 71% among the elderly compared to less than 20% among the 45–54 years age group. More than 80% of respondents aged 75 and over report experiencing some pain or discomfort (extreme in 11% of cases); compared to fewer than 10% among those under 35 years old (0% reported as extreme). Approximately four times as many respondents (over 65%) aged over 65 report experiencing anxiety or depression compared with those below 35 years. The frequency of moderate and severe problems in the “mobility” component also rises with age; the increase between the age groups 55–64 and 65–74 years is especially noticeable, rising from 43.5% to 67.9%. The rarest problems among all age groups are found in the self-care dimension where problems are observed in less than 10% of cases until the age of 54, though increasing sharply thereafter, to more than 40% among those over 65 years.

**Fig 2:**
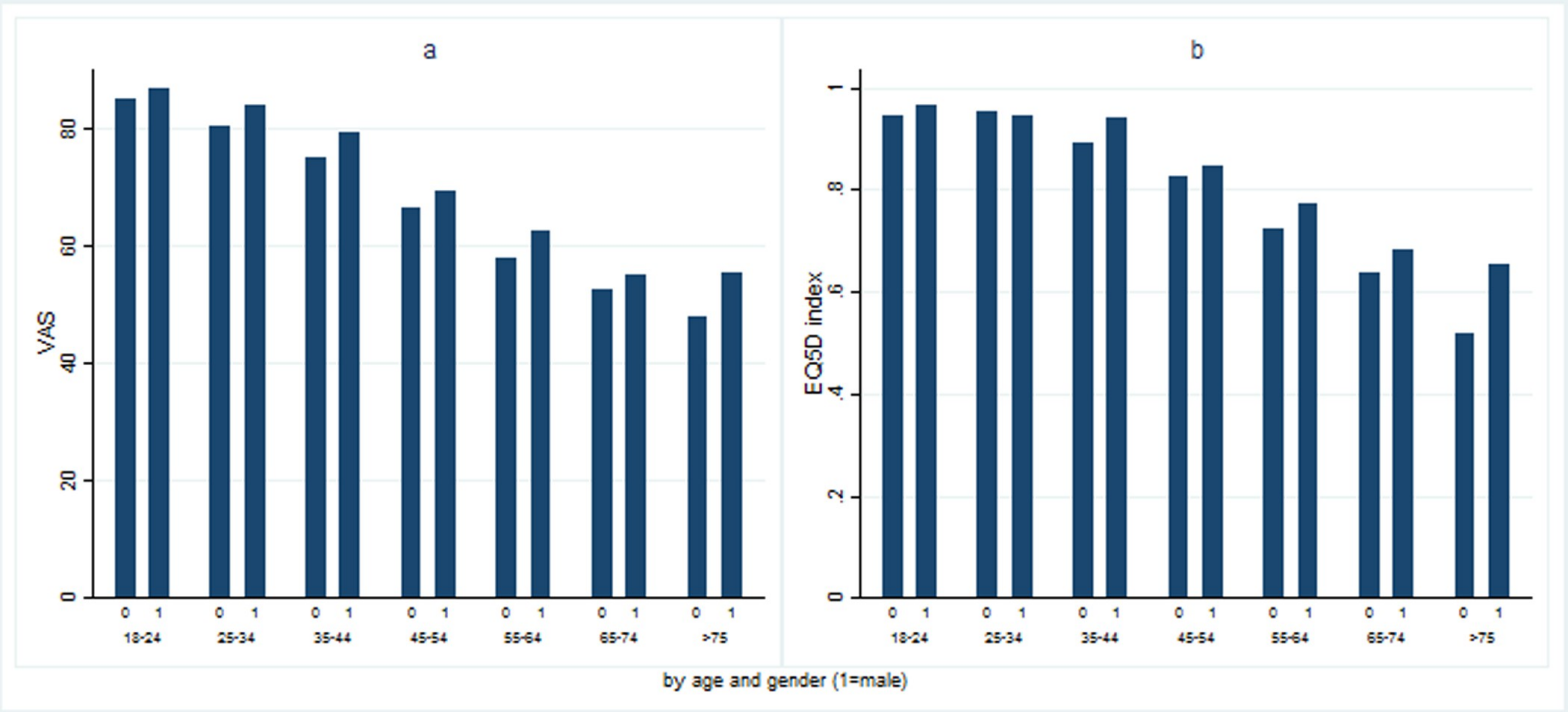
Mean VAS and index scores by age and gender groups. a. VAS score, b. EQ-5D index score.

From the 243 possible EQ-5D-3L health states, the Levada survey returns just 60 different health states for the Russian population, with just over half (52.7%) of the respondents reporting the absence of HRQOL problems in all five dimensions (health state “11111”). The corresponding figures by gender are 60% (males) and 47% (females). From the 60 reported health states, the 15 most common, accounting for 93% of responses, are depicted in Table 5. Health states “11111” (full health), “11112” (moderate problems in anxiety/depression) and “22222” (moderate problems in all five domains) were the most frequent responses in the sample, accounting for 70.3% of overall responses. It is noteworthy that, among the health states listed, there are no severe problems reported in any single dimension. Deviation from a level 1 report (no problems) among the five dimensions gradually increases with age. At the age of 18–24, 85% of respondents describe their health status as “11111”; for those aged 35–44 years, the proportion drops to 67%, before falling further to 31% among those aged 55–64 years (Fig 2).

**Table 5:** Most frequent health states with mean EQ–VAS and utility scores.

EQ–5D states	Number	Cum. %	EQ–VAS^a^		EQ-5D Index
			Mean	95% CI	
**11111**	825	52.7	80.7	79.5; 81.9	1
**11112**	149	62.2	73.7	71.3; 76.1	0.85
**22222**	126	70.3	47.2	44.4; 49.9	0.52
**11122**	83	75.6	61.0	57.5; 64.5	0.73
**21222**	65	79.7	50.3	46.5; 54.2	0.62
**21122**	51	83.0	55.1	52.2; 57.9	0.66
**11121**	33	85.1	68.9	63.8; 74.1	0.80
**21221**	23	86.6	53.7	46.9; 60.6	0.69
**11222**	17	87.7	66.1	58; 74.1	0.69
**21121**	17	88.8	62.1	55.4; 68.7	0.73
**22221**	17	89.8	58.5	47.7; 69.4	0.59
**21111**	16	90.9	67.2	61.5; 72.9	0.85
**22211**	13	91.7	71.2	59; 83.3	0.71
**21112**	11	92.4	59.9	49.5; 70.3	0.78
**11212**	10	93.0	66.2	58.3; 74.1	0.81

Note.

^a^ p-value < 0.0001 (Spearman’s rank correlation analyses) indicating a strong association between the EQ–VAS and the EQ-5D Index score.

Table 5 also shows that the EQ–VAS scores vary significantly according to whether the respondents report any problems in each dimension. Overall, those reporting no problems have an EQ-VAS (EQ-5D Index) score seven (0.15) points higher than those who report at least one problem in any dimension. The correlation between EQ–VAS scores and EQ–5D index was found to be statistically significant and strong (*ρ* = 0.61. p−value<0.0001). Mean VAS (and Index) scores decrease significantly with age (Fig 2), with 61% of men and women below age 35, reporting mean VAS scores above 80, while just 2% of those over 65 years of age report similar scores.

Table 6 presents the odds ratios (OR) for the regression estimates for each dimension and for the basic and augmented specification in each case. The reference population group is women, age 18–24 years, with primary education. Starting with age, it is striking that, beyond the age of 34, each subsequent decade increases the odds substantially of reporting problems across the EQ-5D dimensions (for mobility and self-care the escalation begins after the age of 44). The pain/discomfort dimension has the highest OR, increasing steeply with age, while anxiety and depression have the lowest age gradients. Women are more likely to report health problems than men, however–interestingly–after controlling for the additional health-related characteristics in the augmented regression, gender does *not* significantly affect the likelihood of reporting problems. People with higher education are 2.2 (OR 0.45) times less likely to report problems in the self-care dimension compared with people who have primary education. However, generally, having higher educational attainment does not seem to affect the overall likelihood of registering problems in the EQ–5D dimensions. Being an urban resident reduces the odds ratio for reporting problems in usual activities by 0.5 (OR 0.53) times compared to residence in a rural area but does not have a significant association with reporting on other dimensions. Marital status is also not a significant predictor of reporting HRQOL problems in the EQ-5D dimensions. As expected, poverty status is positively associated with poor health in most dimensions. Respondents reporting frequent feelings of anxiety or blue mood are 3.3 (2.5–4.5. 95% CI) times more likely to report problems in any of five dimensions, while the presence of at least one chronic condition significantly increases the risk of problems in all EQ–5D dimensions except for self-care. Finally, those reporting a disability status are 5.6 times more likely to have problems in the usual activity dimension and 6 times more likely to feel pain/discomfort as well as being more likely to report problems in the remaining dimensions.

**Table 6:** Odds ratios for different socio-economic groups reporting any problems on each EQ–5D dimension.

	Mobility (OR^a^)		Self-care (OR)		Usual activities (OR)		Pain/Discomfort (OR)		Anxiety/Depression (OR)		Problem in any dimension (OR)	
	1^b^	2^c^	3	4	5	6	7	8	9	10	11	12
**Gender**	0.59***	0.82	0.62**	0.72	0.61***	0.75	0.67**	0.91	0.67***	0.84	0.67***	0.84
	[0.44.0.78]^d^	[0.58.1.14]	[0.44.0.87]	[0.47.1.08]	[0.46.0.81]	[0.54.1.06]	[0.52.0.87]	[0.67.1.23]	[0.53.0.84]	[0.65.1.09]	[0.53.0.85]	[0.65.1.09]
**Age group**												
25–34	0.92	0.87	0.75	0.69	1.31	1.15	1.41	1.23	1.40	1.13	1.67	1.48
	[0.36.2.39]	[0.32.2.38]	[0.23.2.44]	[0.20.2.42]	[0.49.3.45]	[0.43.3.05]	[0.61.3.26]	[0.51.2.98]	[0.80.2.45]	[0.62.2.07]	[0.99.2.80]	[0.85.2.57]
35–44	1.69	1.56	1.31	1.21	2.74*	2.34	3.24**	2.91*	2.69***	2.02*	2.87***	2.53**
	[0.69.4.13]	[0.57.4.24]	[0.44.3.88]	[0.35.4.15]	[1.10.6.84]	[0.89.6.15]	[1.47.7.18]	[1.23.6.91]	[1.56.4.63]	[1.12.3.63]	[1.72.4.78]	[1.44.4.44]
45–54	7.10***	5.09***	2.98*	2.23	6.59***	4.19**	11.29***	8.40***	4.99***	2.94***	6.24***	4.34***
	[3.11.16.22]	[1.95.13.29]	[1.08.8.16]	[0.64.7.71]	[2.73.15.94]	[1.59.11.01]	[5.24.24.33]	[3.60.19.60]	[2.90.8.58]	[1.62.5.34]	[3.73.10.45]	[2.44.7.72]
55–64	15.50***	9.72***	8.19***	5.58**	15.16***	8.74***	19.86***	12.42***	8.36***	4.31***	12.32***	7.48***
	[6.93.34.66]	[3.79.24.97]	[3.18.21.10]	[1.73.18.05]	[6.41.35.84]	[3.42.22.32]	[9.31.42.38]	[5.33.28.96]	[4.91.14.23]	[2.38.7.81]	[7.36.20.60]	[4.18.13.37]
65–74	41.26***	21.70***	20.15***	12.22***	28.63***	13.79***	45.16***	23.90***	12.56***	5.634***	28.32***	15.08***
	[17.96.94.75]	[8.00.58.91]	[7.80.52.10]	[3.69.40.49]	[11.91.68.81]	[5.15.36.88]	[20.48.99.58]	[9.69.58.91]	[7.12.22.17]	[2.96.10.73]	[15.52.51.70]	[7.59.29.95]
>75	71.05***	36.59***	31.54***	18.55***	57.28***	30.62***	84.85***	45.20***	11.53***	4.54***	49.73***	25.11***
	[28.15.179.34]	[12.29.108.89]	[11.63.85.53]	[5.16.66.68]	[22.17.148.03]	[10.41.90.08]	[33.75.213.28]	[16.28.125.48]	[6.03.22.08]	[2.04.10.11]	[21.25.116.39]	[9.96.63.32]
**Education level**												
Secondary	0.99	1.09	0.72	0.76	0.67*	0.66*	0.84	0.93	0.85	0.99	1.05	1.27
	[0.71.1.39]	[0.75.1.59]	[0.50.1.03]	[0.51.1.13]	[0.48.0.92]	[0.46.0.95]	[0.61.1.15]	[0.65.1.32]	[0.64.1.11]	[0.73.1.35]	[0.78.1.40]	[0.91.1.76]
Higher	0.60*	0.72	0.45***	0.54*	0.60**	0.74	0.64*	0.82	0.76	0.99	0.85	1.16
	[0.41.0.90]	[0.46.1.12]	[0.28.0.72]	[0.32.0.93]	[0.41.0.89]	[0.49.1.13]	[0.44.0.91]	[0.54.1.24]	[0.56.1.04]	[0.70.1.41]	[0.61.1.17]	[0.81.1.68]
**Marital status**												
Married		0.58		0.62		0.79		0.70		0.99		0.82
		[0.30.1.15]		[0.28.1.38]		[0.43.1.44]		[0.40.1.22]		[0.63.1.57]		[0.53.1.28]
Divorced		0.85		0.73		1.12		0.84		1.17		0.85
		[0.37.1.96]		[0.27.1.94]		[0.52.2.39]		[0.41.1.70]		[0.64.2.13]		[0.46.1.54]
Widowed		1.03		0.78		0.93		1.06		1.156		1.07
		[0.46.2.31]		[0.32.1.92]		[0.44.1.98]		[0.51.2.23]		[0.62.2.17]		[0.54.2.12]
**Not enough money for food and clothes**		1.69**		1.55*		1.39		2.02***		2.06***		2.12***
		[1.16.2.46]		[1.04.2.31]		[0.97.2.00]		[1.42.2.86]		[1.53.2.78]		[1.51.2.98]
**Urban area**		0.73		0.69		0.53***		0.84		0.96		0.81
		[0.50.1.06]		[0.46.1.03]		[0.37.0.76]		[0.59.1.19]		[0.71.1.29]		[0.60.1.09]
**Chronic conditions**												
One chronic condition		2.78***		1.45		1.81**		2.75***		1.587**		2.15***
		[1.92.4.02]		[0.93.2.25]		[1.25.2.60]		[1.94.3.89]		[1.16.2.17]		[1.55.2.99]
Two chronic conditions		4.10***		1.35		2.63***		3.46***		2.35***		3.85***
		[2.38.7.04]		[0.76.2.43]		[1.53.4.50]		[2.03.5.89]		[1.45.3.81]		[2.12.6.96]
Three or more chronic conditions		16.88***		1.60		11.08***		13.61***		3.81***		34.72***
		[7.15.39.87]		[0.75.3.39]		[5.19.23.63]		[5.05.36.67]		[1.86.7.78]		[4.47.269.92]
**Negative feelings (blue mood)**		1.72**		1.98***		1.87***		2.55***		4.08***		3.35***
		[1.22.2.44]		[1.36.2.87]		[1.34.2.61]		[1.85.3.49]		[3.12.5.32]		[2.47.4.53]
**Disability**		4.40***		6.12***		5.60***		6.04***		2.24***		5.29***
		[2.38.8.14]		[3.65.10.24]		[3.29.9.53]		[3.29.11.09]		[1.41.3.55]		[2.59.10.82]

Note.

^a^ OR refers to Odds Ratio

^b^ The first column with the OR in each dimension refers to results adjusted by gender, age and education level

^c^ The second column refers to results adjusted by gender, age, education level and additional socio-demographic characteristics

^d^ 95% confidence intervals in brackets

* p<0.05

** p<0.01

*** p<0.001.

## Discussion

This is the first study to present EQ-5D population norms for the Russian Federation and therefore represents an important advance in HRQOL studies in Russia. Among the main findings are that: (1) the largest proportion of problems reported are in the anxiety/depression dimension; (2) women report more problems in all dimensions than men but, this observed gender effect is attenuated by the inclusion of additional health characteristics in the regressions; (3) problems reported in the EQ–5D dimensions increase dramatically in line with the respondents age; (4) education level, marital status and urban dwelling do not appear to be significant determinants of problems; but (5) chronic diseases, disability and frequent "blue mood" reduce HRQOL scores significantly within the EQ-5D-3L instrument. In Russia, it would seem therefore, that the EQ-5D instrument is appropriately sensitive to underlying observed and unobserved health problems.

The mean EQ-VAS score (70.5) is similar to EQ-VAS scores reported for Hungary (70.4), Korea (71.3) and Argentina (73.9), but much lower than for Denmark (83.3), the UK (82.8) and Sweden (82.5) [31]. Indeed, even comparing the Russian EQ-VAS with other emerging and middle-income economies, such as Argentina (73.9), China (79.9) and Thailand (78.9), the Russian scores appear to be on the low side. The mean EQ–5D Index score is 0.84, which again is lower than for Argentina (0.90), the UK (0.86), the US (0.87) and Korea (0.96) [31]. Since, in the absence of a representative Russian value set, we use the UK utility tariff to score the EQ-5D Index, the comparative results for the latter reflect the combination of Russia specific socio-economic inequalities blended with the population attitudes and perceptions towards health outcomes of the UK population [33].

Despite the overall aggregate differences, that more than half (53%) of the Levada sample report ‘full’ health (11111) is generally consistent with earlier results for Sweden (51%), the UK (58%) and Poland (47%) [34–36]. It may be that the EQ-5D-3L is not sufficiently sensitive to minor health complaints among the population that are generally healthy in Russia [37]. Similarly, the age gradients we report for Russia are in line with other recent results for Poland [38], China [39], England [40], Sweden [34], Denmark [41], Singapore [42], Hungary [43] and Japan [44]. These findings reflect well-established results relating to multiple social and biological processes, the effects of which, accumulate over the life-cycle [45]. These gradients are particularly important to understand in Russia, with its growing elderly population and with a limited tradition in health and social care of sensitivity to age specific health problems.

Beyond age gradients, there are several distinctive features of the Russian norms we present, compared with other countries. First, gender differences in EQ-VAS scores are higher in Russia compared to other European countries, with a six-unit difference between male and female results compared with a high of 4 across Hungary, Italy and Spain [46]. Notwithstanding this difference, logistic models suggest that gender is *not* associated with poor health once other health variables are controlled for. This is consistent with findings for Australia [47], Sri Lanka [48], Sweden [46], Vietnam [49] and, more broadly, for work based on European and US surveys which shows that gender differences in SAH disappear in the majority of cases, after controlling for disease presence and other health problems [50].

Second, whereas usually the most common problems are in the pain/discomfort dimension [46], in Russia the most frequently reported category is that of anxiety/depression, with 43% (31%) of women (men) reporting problems in this dimension. Not only does this speak to the depth and breadth of mental health problems in Russia but, since one third of respondents’ report that they do not take into account their mental health during the EQ-VAS evaluation, it is possible that these scores represent an overestimation of the true HRQOL. This merits further exploration in the context of literature examining HRQOL instruments as tools for evaluation in the presence of widespread mental health problems [51,52].

Third, while many studies provide evidence that HRQOL varies according to education, family status, and behavioural/psychosocial attributes [53–57], we find no such patterns in the regression results based on the Levada data. The absence of a strong education gradient is particularly striking given the well-known strength of the association between health and education. This finding merits further consideration in future research.

Finally, returning to the age gradients, these Russian data suggest that each accumulated decade results in a rapid rise in health problems captured by the pain/discomfort and mobility dimensions whereas, elsewhere, age is more strongly associated with the occurrence of problems in self-care and mobility [43]. As Russians age, the likelihood of their reporting chronic diseases, disabilities, and negative psychological feelings, such as blue mood, anxiety and depression, significantly increases and–of course–this contributes to the changing HRQOL profile over the life cycle. However, we note with interest that, according to our logistic regression results, the presence of chronic disease does not impact on the self-care dimension of HRQOL suggesting, consistent with earlier work [31], that Russian’s are able to adapt (at least on some dimensions) to changes in their health states which are of a persistent nature. This contrasts with results reported elsewhere [57–59].

### Limitations

Notwithstanding the importance of these first Russian population norms, as with all empirical work, there are limitations to this research. First, while these data are nationally representative, Russia is the largest country in the world and is characterised by unparalleled regional heterogeneity, which these data do not allow us to explore. Second, in presenting the EQ-5D Index, we have scored the Russian data using the original UK value set. As a Russian EQ-5D-3L value set has recently been presented [20] future research will re-calibrate the EQ-5D Index using these new population preferences. Third, while we have highlighted several important associations and apparent relationships, given the cross-sectional nature of these data, there is little we can say at this stage, about the underlying causal relationships.

## Conclusion

The EQ-5D instrument facilitates the collection of data on population and/or patient group specific HRQOL which is vital for describing general and specific population well-being. Aside from intra-group and cross-national comparisons, EQ-5D data allows us to understand and analyse the burden of disease and, potentially, to make further clinical and economic decisions concerning the use of health resources. This study presents the first nationally representative Russian population norms for the EQ-5D instrument. The Russian population is shown to follow similar patterns to that seen elsewhere but with several important Russian specific distinctions noted.

We believe that this study represents an important landmark for HRQOL studies in Russia as well as for the prospects of continuing to develop the scholarly and practical infrastructure necessary for Russian Health Technology Assessment to develop. In the first instance, it provides a population reference point for the many clinical and other studies that routinely include the EQ-5D instrument in their data collection. In the longer term though, it paves the way for a broader understanding of EQ-5D among the growing number of users. This provides a positive stimulus for deepening and broadening the community of scholars, policy makers and other stakeholders who are committed to incorporating consideration of HRQOL in clinical and health care decision making.

## Supporting information

S1 Table Profiles of EQ–5D–3L %. (PDF)

S2 Table Profiles of EQ–5D–3L by age groups %. (PDF)

S1 File Original survey questionnaire. (PDF)

S2 File English survey questionnaire. (PDF)

S1 Data Data anonymised. (XLSX)
