# Supplementary material for: First population norms for the EQ-5D-3L in the Russian Federation
Source: PLoS One. 2022 Mar 29;17(3):e0263816. doi: 10.1371/journal.pone.0263816 (PMC8963536; doi:10.1371/journal.pone.0263816)
Supplement: S1 Table — (PDF) [file pone.0263816.s001.pdf]

S1 Table. Profiles of EQ–5D–3L %

Dimension		Levels 1[a]	2	3	Total
**D1**	**Mobility**	1159	393	13	1565
		74.06[b]	*25.11*	*0.83*	*100*
**D2**	**Self-care**	1346	209	10	1565
		86.01	*13.35*	*0.64*	*100*
**D3**	**Usual activity**	1211	340	14	1565
		77.38	*21.73*	*0.89*	*100*
**D4**	**Pain/Discomfort**	1061	478	26	1565
		67.80	*30.54*	*1.66*	*100*
**D5**	**Anxiety/Depression**	979	562	24	1565
		62.56	*35.91*	*1.53*	*100*

Note.
[a]1 – no problems; 2 – moderate problems; 3 – severe problems; [b] the percentage of respondents is in italic.
