# Supplementary material for: First population norms for the EQ-5D-3L in the Russian Federation
Source: PLoS One. 2022 Mar 29;17(3):e0263816. doi: 10.1371/journal.pone.0263816 (PMC8963536; doi:10.1371/journal.pone.0263816)
Supplement: S2 Table — (PDF) [file pone.0263816.s002.pdf]

S2 Table. Profiles of EQ–5D–3L by age groups %

	Dimension	Level	18–24	25–34	35–44	45–54	55–64	65–74	>74
**D1**	**Mobility**	1[a]	95.42	96.08	92.88	74.90	56.54	33.14	21.69
		2	3.92	3.31	6.78	24.70	42.05	65.12	77.11
		3	0.65	0.60	0.34	0.40	1.41	1.74	1.20
**D2**	**Self-care**	1	96.73	97.89	96.27	91.50	78.45	59.30	46.99
		2	2.61	1.81	3.05	8.10	20.85	39.53	51.81
		3	0.65	0.30	0.68	0.40	0.71	1.16	1.20
**D3**	**Usual activity**	1	96.08	95.48	90.85	80.16	62.54	46.51	28.92
		2	3.27	4.52	8.47	19.84	35.34	52.91	66.27
		3	0.65	0.00	0.68	0.00	2.12	0.58	4.82
**D4**	**Pain/Discomfort**	1	94.77	93.37	85.76	62.75	47.70	28.49	16.87
		2	5.23	6.63	13.90	36.03	49.82	68.02	72.29
		3	0.00	0.00	0.34	1.21	2.47	3.49	10.84
**D5**	**Anxiety/Depression**	1	86.93	83.43	72.20	57.89	44.17	34.30	34.94
		2	12.42	16.27	27.12	40.89	53.00	63.95	57.83
		3	0.65	0.30	0.68	1.21	2.83	1.74	7.23

Note.

[a]1 – no problems; 2 – moderate problems; 3 – severe problems.
