# Supplementary material for: First population norms for the EQ-5D-3L in the Russian Federation
Source: PLoS One. 2022 Mar 29;17(3):e0263816. doi: 10.1371/journal.pone.0263816 (PMC8963536; doi:10.1371/journal.pone.0263816)
Supplement: S1 File — (PDF) [file pone.0263816.s003.pdf]

Опросник по качеству жизни связанному со здоровьем в России

I Часть. Социально-демографические вопросы, являющиеся постоянной частью омнибусного опроса

1. Ваш пол:	o Мужской<br>o Женский
2. Ваша дата рождения:	_____._____.______
3. Каково Ваше семейное положение?	o Холост (не замужем), никогда не были в браке<br>o Живете вместе, но не зарегистрированы<br>o Состоите в браке<br>o Официально зарегистрированы, но вместе не живете<br>o Вдовец (вдова)<br>o Разведены и в браке не состоите<br>o Затрудняюсь ответить<br>o Отказ от ответа
4. Какой уровень образования Вы имеете?	o Начальное общее образование – не окончено 8, сейчас 9 классов школы<br>o Основное общее образование – окончено 8, сейчас 9 классов школы<br>o Профессиональные курсы на базе неполного основного общего образования<br>o Среднее (полное) общее - окончено 10, сейчас 11 классов школы<br>o Среднее профессиональное образование (пту, колледж)<br>o Среднее специальное образование (техникум)<br>o Неполное высшее профессиональное образование<br>o Законченное высшее образование и выше<br>o Затрудняюсь ответить<br>o Отказ от ответа
5. Какой ответ лучше всего описывает род Ваших основных	o Предприниматель, самозанятый<br>o Руководитель, управленческий работник<br>o Специалист без руководящих функций

занятий в настоящее время?	(со специальным образованием)<br>○ Рабочий (в том числе мастер, бригадир), в т.ч. в сельском хозяйстве<br>○ Работник сферы услуг за исключением торговли<br>○ Веду домашнее хозяйство, ухаживаю за другими членами семьи, воспитываю ребенка<br>○ Учащийся, студент<br>○ Пенсионер (неработающий) по старости/выслуге лет<br>○ Пенсионер (неработающий) по инвалидности<br>○ Временно не работаю и ищу работу<br>○ Не работаю и не ищу работу<br>○ Затрудняюсь ответить<br>○ Отказ от ответа
6. Сколько всего человек проживает вместе с вами, включая Вас лично?	________________________ человек<br>○ Затрудняюсь ответить<br>○ Отказ от ответа
7. Сколько у Вас детей в возрасте до 16 лет?	○ ________________________ детей<br>○ Нет детей в возрасте до 16 лет<br>○ Затрудняюсь ответить<br>○ Отказ от ответа

8. К какой из следующих групп населения вы скорее могли бы себя отнести?	○ Нам не хватает денег даже на питание<br>○ Нам хватает денег на питание, но не хватает на одежду<br>○ Нам хватает денег на питание и одежду, покупка более дорогих вещей, таких как телевизор и холодильник, вызывает у нас проблемы<br>○ Мы можем покупать некоторые дорогие вещи, такие как холодильник и телевизор, но не можем купить автомобиль<br>○ Мы можем купить автомобиль, но не можем сказать, что не стеснены в средствах<br>○ Мы можем ни в чем себе не отказывать<br>○ Затрудняюсь ответить<br>○ Отказ от ответа
9. Каков был общий доход вашей семьи из всех источников за последние 30 дней?	○ _________________________ рублей<br>○ Затрудняюсь ответить<br>○ Отказ от ответа

II Часть. Специальные вопросы, касающиеся здоровья и качества жизни

1. Хватает ли Вам сил, энергии для обычной жизни?	○ Совсем не хватает<br>○ Едва хватает<br>○ Более менее хватает<br>○ Как правило, хватает<br>○ Всегда хватает- я полон сил<br>○ Затрудняюсь ответить<br>○ Отказ от ответа
2. Есть ли у Вас хронические заболевания? Если да, то какие (несколько ответов):	☐ Нет хронических заболеваний<br>☐ Есть хронические заболевания, но не хочу сообщать какие<br>☐ Заболевания сердца<br>☐ Заболевания легких<br>☐ Заболевания печени<br>☐ Заболевания почек

	☐ Гастроэнтерологические заболевания<br>☐ Аллергия<br>☐ Оториноларингологические заболевания (ЛОР)<br>☐ Рак<br>☐ Заболевания позвоночника<br>☐ Другие хронические заболевания (пожалуйста, укажите какие):<br>_______________________________________<br>☐ Затрудняюсь ответить<br>☐ Отказ от ответа
3. Как часто у Вас бывают такие неприятные чувства, как, например, плохое настроение, уныние, тревога, депрессия?	○ Никогда<br>○ Редко<br>○ Довольно часто<br>○ Очень часто<br>○ Постоянно<br>○ Затрудняюсь ответить<br>○ Отказ от ответа
4. Как Вы в целом оцениваете свое здоровье?	○ Очень хорошее<br>○ Хорошее<br>○ Среднее, ни хорошее, ни плохое<br>○ Плохое<br>○ Очень плохое<br>○ Затрудняюсь ответить<br>○ Отказ от ответа
5. А как бы Вы оценивали свое здоровье по сравнению с людьми Вашего пола и возраста	○ Гораздо лучше, чем в среднем у людей моего пола и возраста<br>○ Скорее лучше, чем в среднем у людей моего пола и возраста<br>○ Примерно такое же, как в среднем у людей моего пола и возраста<br>○ Скорее хуже, чем в среднем у людей моего пола и возраста<br>○ Гораздо хуже, чем в среднем у людей моего пола и возраста<br>○ Затрудняюсь ответить

	○ Отказ от ответа
6. У Вас есть какая-либо группа инвалидности?	○ Нет группы инвалидности<br>○ Раньше была инвалидность, но ее сняли, т.к. здоровье улучшилось<br>○ Раньше была инвалидность, но ее сняли, хотя здоровье не стало лучше<br>○ Я сейчас оформляю инвалидность<br>○ Есть инвалидность 1 группы<br>○ Есть инвалидность 2 группы<br>○ Есть инвалидность 3 группы<br>○ Затрудняюсь ответить<br>○ Отказ от ответа

III Часть. EQ-5D-3L вопросник

Отметьте галочкой ОДИН квадрат в каждом из разделов, приведенных ниже. Укажите такие ответы, которые наилучшим образом отражают состояние Вашего здоровья на СЕГОДНЯШНИЙ ДЕНЬ.

ПОДВИЖНОСТЬ

Я не испытываю трудностей при ходьбе ☐

Я испытываю некоторые трудности при ходьбе ☐

Я прикован (-а) к постели ☐

УХОД ЗА СОБОЙ

Я не испытываю трудностей при уходе за собой ☐

Я испытываю некоторые трудности с мытьем или одеванием ☐

Я не в состоянии сам (-а) мыться или одеваться ☐

ПОВСЕДНЕВНАЯ ДЕЯТЕЛЬНОСТЬ *(например: работа, учеба, работа по дому, участие в делах семьи, досуг)*

Я не испытываю трудностей в моей привычной повседневной деятельности ☐

Я испытываю некоторые трудности в моей привычной повседневной деятельности ☐

Я не в состоянии заниматься своей привычной повседневной деятельностью ☐

БОЛЬ / ДИСКОМФОРТ

Я не испытываю боли или дискомфорта ☐

Я испытываю умеренную боль или дискомфорт ☐

Я испытываю крайне сильную боль или дискомфорт ☐

ТРЕВОГА / ДЕПРЕССИЯ

Я не испытываю тревоги или депрессии ☐

Я испытываю умеренную тревогу или депрессию ☐

Я испытываю крайне сильную тревогу или депрессию ☐

- Мы хотели бы узнать, как Вы оцениваете состояние своего здоровья на СЕГОДНЯШНИЙ ДЕНЬ.
- Перед Вами шкала от 0 до 100.
- 100 означает наилучшее состояние здоровья, которое вы можете представить.

  0 – <u>наихудшее</u> состояние здоровья, которое вы можете представить.
- Поставьте крестик “X” на шкале в том месте, которое, по Вашему мнению, соответствует состоянию Вашего здоровья СЕГОДНЯ.
- Теперь впишите отмеченное Вами на шкале число в приведенный ниже квадрат.

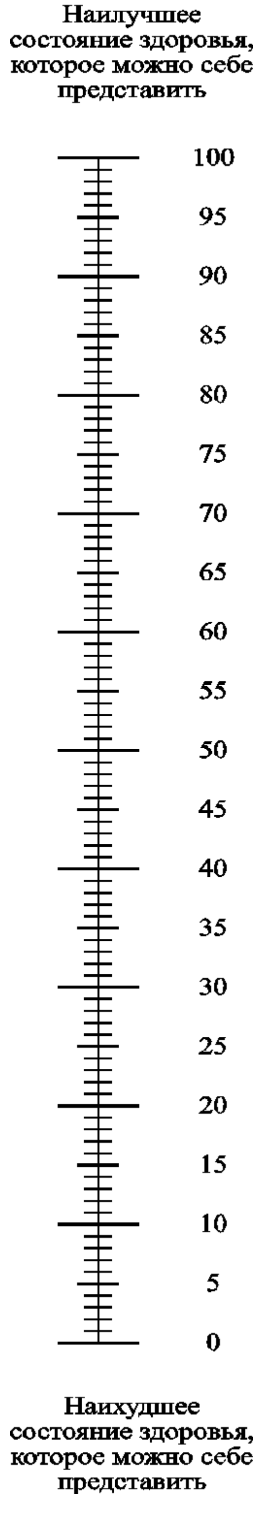

СОСТОЯНИЕ ВАШЕГО ЗДОРОВЬЯ СЕГОДНЯ = ☐

IV Часть. Оценка психического здоровья в общем здоровье.

1. Когда Вы отвечали на предыдущий вопрос и по шкале от 0 до 100 оценили свое здоровье, учитывали ли Вы свое психологическое самочувствие, помимо физического здоровья?	o Да, я учитывал(-а) свое психологическое самочувствие<br>o Нет, я не учитывал(-а) свое психологическое самочувствие<br>o Затрудняюсь ответить<br>o Отказ от ответа
