# Supplementary material for: First population norms for the EQ-5D-3L in the Russian Federation
Source: PLoS One. 2022 Mar 29;17(3):e0263816. doi: 10.1371/journal.pone.0263816 (PMC8963536; doi:10.1371/journal.pone.0263816)
Supplement: S2 File — (PDF) [file pone.0263816.s004.pdf]

Questionnaire on the health-related quality of life in Russia

Part I. Socio-demographic questions included in the general Omnibus Survey

1. Your gender:	○ Male<br>○ Female
2. Date of birth:	_____.______._____
3. What is your marital status?	○ Single<br>○ Living together without registration<br>○ Married<br>○ Married, but living separately<br>○ Widowed<br>○ Divorced<br>○ Does not know<br>○ Refuses to answer
4. What is the highest level of education you completed?	○ Primary general education- not finished 8 grades (9 grades of school with an 11-year school system)<br>○ Middle school – finished 8 grades (9 grades of school with an 11-year school system)<br>○ Vocational training based on incomplete middle school<br>○ Secondary school diploma - finished 10 grades (or 11 grades of school now)<br>○ Secondary / vocational training based on complete middle school<br>○ Secondary special education / technikum<br>○ Unfinished higher education<br>○ Higher education diploma and more<br>○ Does not know<br>○ Refuses to answer
5. Which answer best describes your main occupation?	○ Entrepreneur, self-employed<br>○ Executive, manager<br>○ White-collar worker<br>○ Blue-collar worker<br>○ Worker in a service sector, but not mongering

	○ A housewife, caring for other family members, raising children<br>○ Student<br>○ Retired and not working<br>○ Unable to work for health reasons, disabled<br>○ Temporarily not employed and looking for a job<br>○ Unemployed and not looking for a job<br>○ Does not know<br>○ Refuses to answer
6. Including yourself, how many people live in your household?	○ ____________________ people<br>○ Does not know<br>○ Refuses to answer
7. How many children under the age of 16 do you have?	○ ____________________ children<br>○ Does not have children under the age of 16<br>○ Does not know<br>○ Refuses to answer
8. Which of the following population groups would you most likely identify yourself with?	○ We have not enough money even for food<br>○ We have enough money for food, but not for clothes<br>○ We have enough money for food and clothes, but it is a problem for us to purchase TV or fridge<br>○ We can purchase some expensive things, such as TV or fridge, but cannot purchase a car<br>○ We can buy a car, but we cannot say that we are not limited in means<br>○ We can deny ourselves nothing<br>○ Does not know<br>○ Refuses to answer
9. What was the total income of your entire family including all the money received in the last	○ ____________________ rubles<br>○ Does not know<br>○ Refuses to answer

30 days?	

Part II. Specific health-related and quality of life questions

1. Do you have enough energy for everyday life?	○ Not at all<br>○ A little<br>○ Moderately<br>○ Mostly<br>○ Completely<br>○ Does not know<br>○ Refuses to answer
2. Do you have any chronic illnesses? If yes, which of the following illnesses do you have (multiple responses possible):	☐ I do not have any chronic illnesses<br>☐ I have a chronic illness but do not want to specify<br>☐ Heart disease<br>☐ Lung disease<br>☐ Liver disease<br>☐ Kidney disease<br>☐ Gastrointestinal disease<br>☐ Allergy<br>☐ Otorhinolaryngology<br>☐ Cancer<br>☐ Spinal problems<br>☐ Other chronic illnesses (please, specify): ______________________<br>☐ Does not know<br>☐ Refuses to answer
3. How often do you have negative feelings such as blue mood, despair, anxiety, depression?	○ Never<br>○ Seldom<br>○ Quite often<br>○ Very often<br>○ Always<br>○ Does not know<br>○ Refuses to answer
4. In general, how would you describe your health?	○ Very good<br>○ Good<br>○ Average, not good, not bad

	○ Bad<br>○ Very bad<br>○ Does not know<br>○ Refuses to answer
5. Compared to the health of same-age and gender peer how would you define your health?	○ Much better than same age and gender peer<br>○ Rather better than same age and gender peer<br>○ The same as have same age and gender peer<br>○ Rather worse than same age and gender peer<br>○ Much worse than same age and gender peer<br>○ Does not know<br>○ Refuses to answer
6. Do you have any disability group?	○ Do not have any disability group<br>○ Had disability group, but disability group was withdrawn due to the health improvement<br>○ Had disability group, but disability group was withdrawn without the health improvement<br>○ I draw up documents to receive the disability group<br>○ First group<br>○ Second group<br>○ Third group<br>○ Does not know<br>○ Refuses to answer

Part III. EQ-5D-3L questionnaire

Under each heading, please tick the ONE box that best describes your health TODAY.

MOBILITY

I have no problems in walking about ☐
I have some problems in walking about ☐
I am confined to bed ☐

SELF-CARE

I have no problems with self-care ☐
I have some problems washing or dressing myself ☐
I am unable to wash or dress myself ☐

USUAL ACTIVITIES *(e.g. work, study, housework, family or leisure activities)*

I have no problems with performing my usual activities ☐
I have some problems with performing my usual activities ☐
I am unable to perform my usual activities ☐

PAIN / DISCOMFORT

I have no pain or discomfort ☐
I have moderate pain or discomfort ☐
I have extreme pain or discomfort ☐

ANXIETY / DEPRESSION

I am not anxious or depressed ☐
I am moderately anxious or depressed ☐
I am extremely anxious or depressed ☐

- We would like to know how good or bad your health is TODAY.
- This scale is numbered from 0 to 100.
- 100 means the best health you can imagine.

  0 means the worst health you can imagine.
- Please mark an X on the scale to indicate how your health is TODAY.
- Now, write the number you marked on the scale in the box below.

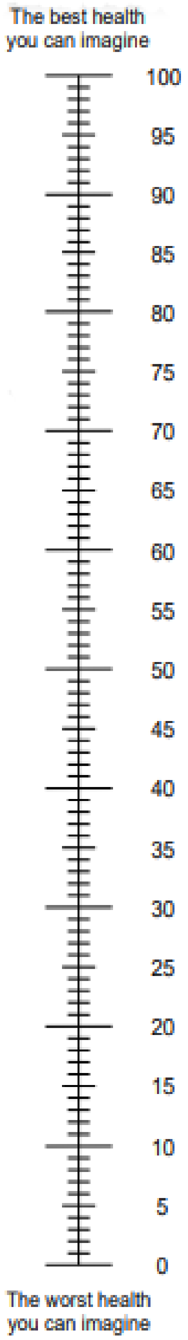

YOUR HEALTH TODAY = ☐

Part IV. Assessment mental health in the general health

7. In answering the previous question and rating your health today on a scale of 0 to 100, did you consider your mental well-being in addition to your physical health?	○ Yes, I considered my mental well-being<br>○ No, I did not consider my mental well-being<br>○ Does not know<br>○ Refuses to answer
